# Interactions between a Trawl Fishery and Spatial Closures for Biodiversity Conservation in the Great Barrier Reef World Heritage Area, Australia

**DOI:** 10.1371/journal.pone.0021094

**Published:** 2011-06-13

**Authors:** Alana Grech, Rob Coles

**Affiliations:** 1 Fisheries Queensland, Department of Employment, Economic Development and Innovation, Cairns, Queensland, Australia; 2 Australian Research Council Centre of Excellence for Coral Reef Studies, James Cook University, Townsville, Queensland, Australia; Biodiversity Insitute of Ontario - University of Guelph, Canada

## Abstract

**Background:**

The Queensland East Coast Otter Trawl Fishery (ECOTF) for penaeid shrimp fishes within Australia's Great Barrier Reef World Heritage Area (GBRWHA). The past decade has seen the implementation of conservation and fisheries management strategies to reduce the impact of the ECOTF on the seabed and improve biodiversity conservation. New information from electronic vessel location monitoring systems (VMS) provides an opportunity to review the interactions between the ECOTF and spatial closures for biodiversity conservation.

**Methodology and Results:**

We used fishing metrics and spatial information on the distribution of closures and modelled VMS data in a geographical information system (GIS) to assess change in effort of the trawl fishery from 2001–2009 and to quantify the exposure of 70 reef, non-reef and deep water bioregions to trawl fishing. The number of trawlers and the number of days fished almost halved between 2001 and 2009 and new spatial closures introduced in 2004 reduced the area zoned available for trawl fishing by 33%. However, we found that there was only a relatively minor change in the spatial footprint of the fishery as a result of new spatial closures. Non-reef bioregions benefited the most from new spatial closures followed by deep and reef bioregions.

**Conclusions/Significance:**

Although the catch of non target species remains an issue of concern for fisheries management, the small spatial footprint of the ECOTF relative to the size of the GBRWHA means that the impact on benthic habitats is likely to be negligible. The decline in effort as a result of fishing industry structural adjustment, increasing variable costs and business decisions of fishers is likely to continue a trend to fish only in the most productive areas. This will provide protection for most benthic habitats without any further legislative or management intervention.

## Introduction

Overfishing and the damage to benthic habitats by activities such as bottom trawl fishing are considered to be one of the greatest threats to marine species and ecosystems globally [1], [2]. Almost 75% of continental shelfs across the world are trawled every year [3] and trawl fishing inevitably leads to physical, biological and chemical effects on the seafloor [4]. The effective management of trawl fisheries requires information on the drivers that influence the spatial distribution of fishing effort [5]. Spatial constraints on the distribution of fishing effort include topographic features (e.g. reefs) and spatial and temporal closures to fishing (e.g. marine reserves; [6], [7]. Decisions on where and when to fish are also influenced by fisheries management strategies, adoption of new technologies (i.e. sonar, global positioning systems and computer mapping [8]), and business rationalisation in response to economic circumstances.

**Figure 1:**
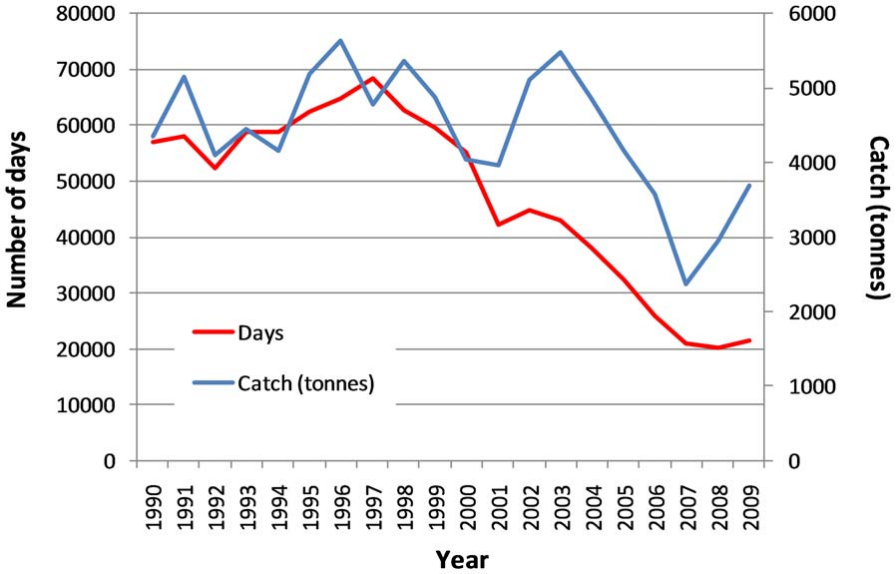
Number of days fished per year by the East Coast Otter Trawl Fishery (ECOTF) and annual trawl catch. The graph shows that the number of days fished in the ECOTF has been in decline since 1997. Despite this reduction in the number of days fished, catch has remained relatively constant with a downward trend beginning in 2004 showing recovery in recent years.

The East Coast Otter Trawl Fishery (ECOTF) of Queensland, Australia fishes for penaeid shrimps (e.g. eastern king prawn and brown tiger prawn) and scallops [9], and is the only demersal fishery in these waters. Depletion trends led to the implementation of multiple management tools in the early 1990's which ensure sustainability, reduce impact on bottom habitats and limit by-catch in the fishery [9], [10]. These management tools include: mandatory turtle excluder devices; restrictions on the number of vessels and their length; limited entry to the fishery; effort reduction strategies; and, spatial and temporal closures that control the spatial footprint of the fishery. Spatial closures are summarised in the Queensland *Fisheries (East Coast Trawl) Management Plan* 1999 and include a mixture of large areas that influence the location and time of fishing effort, and small and complex closures that are specifically targeted (e.g. scallop replenishment sites and ports). Temporal closures are employed widely in the ECOTF to synchronise the fishery with times when the target species are of an optimal size to capture highest market value. In addition, the northern two thirds of the fishery underwent a dramatic change in 2004 with the introduction of an ecosystem-scale network of ‘no take’ marine reserves (Figure 1) covering ∼33% of the Great Barrier Reef World Heritage Area (GBRWHA) [11], [12]. The goal of this network is to improve biodiversity conservation through a comprehensive and representative multiple-use zoning regime [13]. The biophysical operational principles designed to achieve the ecological objectives of the new zoning included specific recommendations to protect at least 20% of the area of 70 reef, non-reef and deep bioregions in ‘no take’ zones [11].

The consequence on catch and effort in the fishery and the change in spatial distribution of trawl fishing resulting from new spatial closures in the GBRWHA has not previously been assessed. Spatial and regulatory complexities and the absence of effort distribution data with the appropriate resolution made an analysis difficult. New spatial information from electronic vessel location monitoring systems (VMS) [14] provides an opportunity to assess change in the fishery over time, and to explore interactions between the ECOTF and spatial closures for biodiversity conservation. In this paper, we: summarise catch and effort trends in the ECOTF over time; quantify change in the spatial footprint of the trawl fishery; and, assess the protection afforded to marine bioregions. We discuss our results in the context of the simultaneous effect of multiple fisheries management strategies on the ECOTF, and the need for precise fisheries catch, effort and fishing location metrics.

## Methods

### Spatial closures

The sources of information on spatial closures of the ECOTF used in our analyses were: the State (Queensland) *Fisheries (East Coast Trawl) Management Plan* 1999; *Great Barrier Reef Coast Marine Park Zoning Plan* 2004; *Fisheries Act* 1994 and its associated regulations; and the Commonwealth (Australian) *Great Barrier Reef Marine Park Zoning Plan* 2003. We obtained geographical information system (GIS) layers on the distribution of spatial closures from the Great Barrier Reef Marine Park Authority, Fisheries Queensland and the Queensland Department of Environment and Resource Management. GIS-layers on the extent of ports was obtained from the relevant port authorities. We derived a single coverage of spatial closures by using the intersect tool in ArcGIS® 9.1 (Environmental Systems Research Institute 2005) to combine GIS-layers of the multiple legislative boundaries. We used the composite coverage of spatial closures to calculate the area zoned available to trawl fishing before and after new spatial closures were introduced in 2004.

### Catch, effort and the spatial distribution of the ECOTF

The large size of the GBRWHA (348,000 km^2^) limits the number, precision and type of catch and effort data that can be collected. Much of the GBRWHA coastline is undeveloped and fishing effort location is constrained by the logistics of distance from a port. Vessels may unload catch at sea and often in remote locations making data collection and verification difficult. Because of these constraints and complexities, Fisheries Queensland adopts a two pronged approach to collecting information on catch and effort in the ECOTF, self reporting for fishing catches, and a satellite based transponder VMS to provide independent effort location mapping.

The self reported retained catch and effort data is collected via compulsory daily fishing logbooks completed by fishers and collated in the Commercial Fisheries Information System (CFISH) database. The information recorded in these logbooks includes: daily retained catch (weight and species); locations fished; and the time spent fishing. The logbook information is aggregated into grids of resolution 6 minutes by 6 minutes. Fisheries Queensland provided collated data on the number of days fished, total catch (in tonnes) and number of licensed vessels in the GBRWHA for each year from 1990 to 2009.

VMS is a satellite-based positional tracking system for monitoring the locations of fishing vessels and is primarily used for enforcement and assessing trends in the fishery. Vessels that operate in the ECOTF are required by law to have an operating VMS transponder on board. Peel and Good [14] used raw VMS position information and logbook catch record data to statistically model the spatial distribution of the ECOTF. Decision rules and techniques were developed by Peel and Good [14] to determine when a vessel was trawling (‘trawl signature’) as intervals of polling frequency vary depending on location. The data was also corrected for known non-fishing times and locations. Modelled layers of the spatial distribution of the ECOTF in the GBRWHA based on VMS data are available for each year from 2001 to 2009 (Figures S1, S2, S3, S4, S5, S6, S7, S8, 9). The layers have a resolution of 1 minute by 1 minute (∼3.61 km^2^) and each grid cell contains estimations of the total number of hours trawled (per year), the total number of boats that trawled within the cell and the total catch (in tonnes).

We used the layers of Peel and Good [14] to estimate the total area trawled between 2001 and 2009 and the amount of area trawled within five time density groups (<5, 5–15, 15–50, 50–100 and >100 hours). Vessels in the trawl fishery use trawl shot lengths of 1 to 4 hours depending on location and the quantity of non target species. We have assumed for the present analysis that based on a maximum likely trawl shot length of four hours [14] recordings of less than five hours per year in a grid is equivalent in impact to one trawl shot.

### Evaluating interactions between marine bioregions and the ECOTF

We assessed the exposure of bottom habitats to trawl fishing using marine bioregions information that was developed during the classification phase of the Representative Areas Program [12]. The Great Barrier Reef Marine Park Authority mapped the biological and physical diversity of the GBRWHA using information from a panel of experts and the best scientific data available at the time. Each bioregion represents an area of known physical features and animal and plant assemblages that are sufficiently distinct from adjacent areas (at the scale of hundreds of kilometres). The experts identified and mapped 70 distinct bioregions (http://kurrawa.gbrmpa.gov.au/corp_site/info_services/publications/sotr/facts.html) and categorised each bioregion as a reef (regions close to or including coral reefs and coral substrates), non-reef (regions of open soft bottom remote from coral structures) or deep (offshore areas that extend from the edge of the continental shelf to the eastern border of the GBRWHA). We overlayed the bioregion layer with the composite coverage of spatial closures and spatial information on trawl distribution derived from VMS data (2001–2009) to estimate the exposure of individual bioregions and groups of bioregions (i.e. reef, non-reef and deep) to trawl fishing.

## Results

Between 1990 and 2009, the number of licensed vessels in the fishery declined by almost 65% (Table 1). In the 2009 fishing season, only 218 vessels were recorded fishing in the GBRWHA (Table 1). The number of days fished between 1990 and 2009 has fluctuated over time; effort increased to a peak of 68,359 days in 1997 and then declined to 21,574 days in 2009 (Table 1; Figure 1). Despite this reduction in vessel numbers and the number of days fished, catch has remained relatively constant with a downward trend after 2004 but recovering in recent years (Table 1; Figure 1).

**Table 1:** Catch and effort statistics for the East Coast Otter Trawl Fishery (ECOTF) in the Great Barrier Reef World Heritage Area (GBRWHA).

Year	Number of vessels	Catch (tonnes)	Number of days fished	Area trawled (km^2^)	Area trawled more than once (km^2^)
1990	593	4463	57115	-	-
1991	623	5275	58034	-	-
1992	566	4272	52414	-	-
1993	509	4570	58804	-	-
1994	487	4262	58781	-	-
1995	489	5292	62409	-	-
1996	504	5808	64897	-	-
1997	509	4940	68359	-	-
1998	476	5497	62835	-	-
1999	454	4986	59661	-	-
2000	505	4184	55239	-	-
2001	408	4037	42284	79109	32195
2002	390	5191	44814	76866	31762
2003	377	5545	42960	74953	29791
2004	355	4901	37990	72857	28382
2005	316	4181	32300	59568	24408
2006	282	3598	25872	54053	22002
2007	239	2384	21050	55635	21774
2008	210	2971	20255	53199	21444
2009	218	3704	21574	54274	22082

Data on the number of vessels, catch and number of days fished was collated by Fisheries Queensland.

**Figure 2:**
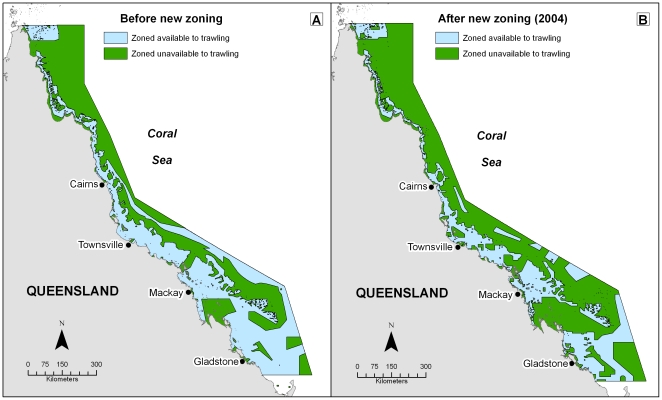
Area zoned available and unavailable to trawling before and after new zoning was introduced in 2004. Almost 51% of the Great Barrier Reef World Heritage Area (177,732 km^2^; GBRWHA) was zoned available to trawl fishing prior to the introduction of new spatial closures in 2004. New zoning decreased the amount of area zoned available to trawl fishing by 59,244 km^2^, and 34% (118,488 km^2^) of the GBRWHA is currently zoned available to trawl fishing.

We found that 51% of the GBRWHA (177,732 km^2^) was zoned available to trawl fishing prior to the introduction of new spatial closures in 2004 (Figure 2). New zoning decreased the amount of area zoned available to trawl fishing by 59,244 km^2^, and 34% (118,488 km^2^) of the GBRWHA is currently zoned available to trawl fishing (Figure 2).

**Figure 3:**
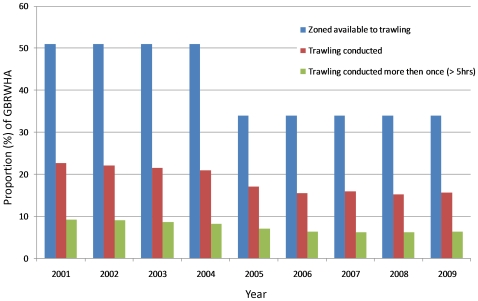
Proportion (%) of the Great Barrier Reef World Heritage Area (GBRWHA) zoned available to trawl fishing, and the proportion of the GBRWHA where trawl fishing was conducted and conducted more than once (i.e. > five hours per year) from 2001–2009. Less than half of the area available for trawling in the GBRWHA was actually trawled between 2001 and 2009. The spatial footprint of the ECOTF steadily declined from 2001–2009 by almost 25,000 km^2^. Most of this decline occurred in areas that were only fished once per year (i.e. < five hours per year). Less than half of the total area trawled in any of the fishing years between 2001 and 2009 was trawled more than once in a year.

Not all areas zoned available for trawling are suitable for trawling due to the complexities of topography and the location of target species. We found that since 2001, trawl fishing has occurred in less than half of the area zoned available for trawling in the GBRWHA (Table 1; Figure 3). In 2001, 23% (∼80,000 km^2^) of the GBRWHA was zoned available for trawling and trawled. The spatial footprint of the fishery has declined slowly and in 2009 only 15% of the GBRWHA was trawled. The largest decline in any one year (approximately 5%) occurred between 2004 and 2005 (Table 1; Figure 3), the same years as the introduction of new spatial closures.

We found that more than half of the total area trawled in any of the fishing years between 2001 and 2009 was trawled only once per year (Figure 3), and on average just ∼450 km^2^ (∼0.2%) of the GBRWHA was trawled for more than 100 hours per year. Many of the areas where new spatial closures were introduced in 2004 cannot have been suitable for trawling or were not trawled regularly. Only 16,642 km^2^ (∼4.8%) of the GBRWHA that was trawled between 2001 and 2004 became zoned unavailable to the ECOTF as a result of new spatial closures. Of the area that became unavailable, most (>83%) was fished only once per year (i.e. < five hours per year).

**Figure 4:**
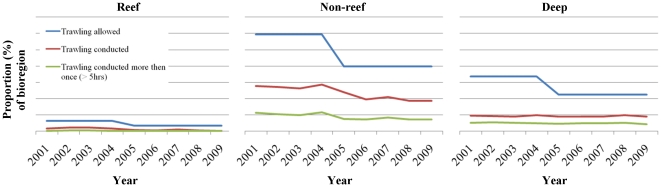
Proportion (%) of the reef, non-reef and deep bioregions of the Great Barrier Reef World Heritage Area (GBRWHA) zoned available to trawl fishing, and the proportion of reef, non-reef and deep bioregions where trawl fishing was conducted and conducted more than once (i.e. > five hours per year) from 2001–2009. Trawl fishing predominantly occurred in non-reef habitats; 18.5–28.6% of non-reef bioregions were trawled from 2001–2009 (Table S1). Non-reef bioregions had the largest reduction of area zoned available for trawl fishing after new spatial closures were introduced in 2004, followed by the deep and reef bioregions (Table S1). Less than half of the area zoned available for trawl fishing in non-reef, reef and deep bioregions was actually trawled from 2001–2009.

We found that between 2001 and 2009, trawl fishing predominantly occurred in non-reef bioregions and 23 of the 32 non-reef bioregions were trawled in 2009 (Table S1). Non-reef bioregions had the largest reduction of area zoned available for trawl fishing (Table S1; Figure 4) after new spatial closures were introduced in 2004 (49,182 km^2^; 19.8% of non-reef bioregions). This was followed by the deep bioregions (9,099 km^2^, 11.3%) and reef bioregions (613 km^2^, 3.1%). 27.7% of non-reef bioregions were trawled in 2001, declining to 18.5% in 2009. There was little change in the area of deep bioregions trawled between 2001 and 2009 (9.5% down to 8.9%) and only 3 of the 8 deep bioregions were exposed to trawling in 2009 (Table S1). 1.5% of reef bioregions were trawled in 2001 declining to 0.2% in 2009. Bioregions that experienced the greatest decline in the proportion of area trawled between 2001–2009 included the central open lagoon reefs (59.8%–0.0%), inner shelf seagrass (81.3%–45.2%), inner mid shelf lagoon (88.0–57.7%) and the coastal southern fringing reefs (30.4–1.0%; Table S1). Bioregions that had >40% of their total area trawled in 2009 were all non-reef (inshore muddy lagoon, inner shelf seagrass, inner mid shelf lagoon and the Capricorn Bunker lagoon; Table S1).

## Discussion

We used fishing metrics and spatial information on the distribution of spatial closures and modelled VMS data to assess change in the spatial distribution of the ECOTF in the GBRWHA. The number of trawlers and the number of days fished almost halved between 2001 and 2009 and new spatial closures introduced in 2004 reduced the area zoned available for trawl fishing by 33%. However, we found that the location of the fishing grounds where most fishing effort is expended did not change markedly between 2001 and 2009. Most of the areas that became unavailable to trawling after new zoning was introduced in 2004 were not trawled or not trawled regularly (<5 hours per year). The large increase in spatial closures resulted in a minimal change in the spatial footprint of the ECOTF, achieving the biophysical objectives of the new zoning [11] with limited socio-economic impact.

The drivers of the spatial footprint of the ECOTF in the GBRWHA are complex. They include a variety of fisheries management tools and marine park management zoning (such as spatial closures for biodiversity conservation) and individual decisions of fishers in response to economic and business circumstances. This is superimposed on an underlying topography, bottom habitat, and the location of target species, which determines where it is possible or desirable to fish. In this paper we found that the long term outcome has been a consistent decline in the potential impact on marine biodiversity and an essentially stable pattern of fishing.

Spatial and temporal closures that affect the ECOTF are complex with a mixture of Commonwealth (Australian), State (Queensland), and local legislation, designated marine parks, port authorities and the World Heritage Area. While the closure complexity makes fisheries management, compliance and enforcement difficult [15] it has had the effect of constraining the fishing grounds. The natural topography also constrains the spatial footprint of the fishery. The long thin shape of the coastline, ∼2,500 km north to south but only 400 km from the coast to the outer trawl grounds at the widest point (and much narrower in most locations) forms overall broad-scale spatial limits . The effect of this is that the trawl fishing grounds also stretch in a long north south strip and effort is clumped in the east - west direction (Figures S1, S2, S3, S4, S5, S6, S7, S8, S9). On the outer reef slopes this is accentuated by topography and the depth range favoured by the target shrimp species. Trawl fishing along a constant depth contour favours a narrow north south trawl pattern. Trawl fishing in the northern half of the GBRWHA is restricted almost entirely to a narrow coastal strip interrupted only by the reef systems and by zoning restrictions. South of this region, effort continues in a coastal strip but is joined with a mid-shelf strip south to Mackay. Between Rockhampton and the southern border of the GBRWHA there is a large mid shelf fishery and a fishery that follows the outer edge of the Swains reefs. The result of this topographic forcing is a fishing pattern that has been resilient to the changes in the area zoned available for trawling with the pattern of heavily fished areas similar across years (Figures S1, S2, S3, S4, S5, S6, S7, S8, S9). The spatial and temporal complexities and constraints effectively limit any potential for the trawl fishery to expand its ecological footprint and are a price paid by the ECOTF for fishing in a complex topography and for the social acceptance of continued fishing in a World Heritage Area.

The total trawled area and number of days fished have been decreasing slowly in response to fisheries management and marine park management. Economic circumstances (high Australian dollar and high fuel costs) also focus trawl fishing at times and in areas historically known to produce higher catch rates. We found that trawl fishing now occurs in less than half the area zoned available to the ECOTF, and only 48% of the available fishing time is used [9]. It is unlikely this trend will reverse as there is are no new licences to fish available and effort in the fishery is capped. These trends combined with the spatial consistency of the fishery through time will ensure any impact of trawl fishing in this sensitive environment remains in its current low form and unlikely to have a negative impact on GBRWHA seabed habitats [16].

Pitcher et al. [10] similarly found little trawl fishing influence on habitat assemblages; little more than expected by chance; and only one species out of 840 analysed exceeded a sustainability indicator reference point. However, Pitcher et al. [10] did not analyse the potential impact of the ECOTF on deep water habitats and there is not sufficient information to conclude that the impact of trawl fishing on these habitats is negligible. In our analyses we found that the spatial footprint of the ECTOF is relatively low in deep water bioregions (Table S1), except the ‘southern embayment’ where 23.5% of the bioregion was trawled in 2009. More research on species assemblages and the potential impact of the trawl fishery on bottom habitats is required in deep water bioregions. In addition, the ECOTF continues to have interactions with species of conservation concern (e.g. some sea snakes and elasmobranch), and further improvements are required to move towards best practice for by-catch reduction.

The overall picture is of a fishing fleet responding to management changes by refining existing trawl fishing locations to maximise catch rather than looking for new or alternative fishing grounds. Shifting trawl fishing effort away from reef bioregions with high biodiversity values while minimising the impact on the overall catch of the fishery confirms the effectiveness of the broad-scale marine spatial planning initiative implemented by the Commonwealth (Australian) Government and complemented by State (Queensland) marine parks legislation [11], [13]. It met biophysical objectives without compromising the socioeconomic constraint to “minimise conflict with commercial extractive users” which the initiative was required to consider and there is evidence of some positive outcomes for the fishery with an increase in CPUE [8], [9].

### Data considerations

Implementation of the VMS system was primarily designed as an enforcement tool in response to the rising cost of boat and aircraft based surveillance for remote fishing grounds and complex zonings [14]. The VMS system polls the vessel location automatically and is independent of the operator. However the VMS location data are relatively unsophisticated –– the transponder provides only a position with no speed, activity or direction of travel information. These are inferred by comparing sequential poll locations. VMS data that we used in our analysis have been filtered and modelled [14] but it is possible that errors remain. At a fishery scale these errors are not likely to affect decision making but if the biological processes that need protecting occur at very small scales (i.e. less than a kilometre), then the spatial resolution of the data presently available would not be appropriate.

The modelled VMS and fisheries logbook data used in our analysis does not include vessel location when the vessel is stationary or when steaming between fishing grounds [14]. Agreements with fishers on access to the fisheries logbook data preclude the use of individual records and use a relatively coarse grid cell to protect private information relating to individual fishing grounds. However, from a GBRWHA management point of view, even with these limitations there is now a spatial record of trawl fishing effort that is accurate and reliable at the scale of the entire GBRWHA. For the first time there is now the ability to estimate the spatial footprint of trawl fishing in the GBRWHA and to follow the response of fishing behaviour to management changes. It is also possible to estimate the level and extent of change in the gross impact of trawl fishing on the bioregions identified during the Great Barrier Reef Marine Park re-zoning process.

The two raw un-modelled spatial data sets (VMS tracking data and catch information) that were available for our analysis are difficult to compare. They are collected for different purposes (enforcement and catch estimation) and in different spatial units. For a fine scale spatial assessment, the CFISH six minute catch data are of limited value and overestimate the actual area from which the catch is taken. The bioregions layer are actual vector shape files not grid cells. A standardised cell/grid/site system for all spatial fisheries and seabed habitat biophysical data would make spatial analysis and the interpretation of the impact of the fishing fleet more precise. Technological solutions such as electronic fishing log books could also be used to collect high resolution catch and effort data that may empirically validate the low resolution commercial data that are currently available. It is possible in the present data base to have catch recorded from self reporting log books in locations where VMS data is not evident. Good et al. [17] recommend ongoing monitoring or validation of fishing catches as part of a long term monitoring program by independent on-board fisheries observers. Financial costs limit the independent on board catch validation by observers that occurs at present. While deficiencies in the data may complicate scientific interpretation they are not as important at a GBRWHA spatial scale of fisheries management. Improving the data available is unlikely to further assist decisions at a precision that it is practical for management.

We have chosen to analyse the fishery at the spatial scale of its management. Sub scale analyses and the spatial vulnerability of individual species to trawl fishing (there are non target species catch concerns for the fishery) is beyond the scope of the present data. However, unless there is an increase in fishing effort, sub-scale spatial analyses are unlikely to add useful information for biodiversity protection. The greatest value to be derived from improved technological solutions to data collection are a more nuanced understanding on the way fishing fleets respond to management interventions. An improved understanding of the response of fishing fleets to management interventions would have avoided some of the problems associated with the Australian government's assistance program in response to changes in the zoning of the GBRWHA [18].

### Conclusions

Trawl fishing effort in the ECOTF is highly clumped in space. Most areas of the GBRWHA are not fished or fished very little. Because of this, at a scale of the whole fishery, the reduction in the area available to fishing that occurred in 2004 removed latent spatial effort but had only a small impact on the amount or pattern of fishing. With a highly clumped fishery such as the ECOTF, removing areas from the fishery has little effect on reducing impact on the bottom if the area chosen is not regularly trawled. Removing areas heavily fished would reduce effort in the fishery but may not provide any greater protection for seabed habitats and benthic species identified as important to protect. Trawl fishing intensity is low at the scale of the GBRWHA with few areas trawled more than a couple of times a year.

The decline in effort in the fishery is likely to continue a trend to fish only in the most productive areas. This provides effective protection to most fishing grounds without any further legislative or management intervention. Satellite based VMS, and compulsory retained catch fishing log books provide for the first time a way of tracking change in response to management intervention at the scale of the whole GBRWHA and a satellite based system for monitoring fishing effort would be desirable for any fishery operating in sensitive marine environments.

## Supporting Information

Figure S1 **Trawl fishing effort (number of hours trawled per year) in 2001** [**17**] **.**
(TIF)

Figure S2 **Trawl fishing effort (number of hours trawled per year) in 2002** [**17**] **.**
(TIF)

Figure S3 **Trawl fishing effort (number of hours trawled per year) in 2003** [**17**] **.**
(TIF)

Figure S4 **Trawl fishing effort (number of hours trawled per year) in 2004** [**17**] **.**
(TIF)

Figure S5 **Trawl fishing effort (number of hours trawled per year) in 2005** [**17**] **.**
(TIF)

Figure S6 **Trawl fishing effort (number of hours trawled per year) in 2006** [**17**] **.**
(TIF)

Figure S7 **Trawl fishing effort (number of hours trawled per year) in 2007** [**17**] **.**
(TIF)

Figure S8 **Trawl fishing effort (number of hours trawled per year) in 2008** [**17**] **.**
(TIF)

Figure S9 **Trawl fishing effort (number of hours trawled per year) in 2009** [**17**] **.**
(TIF)

Table S1 Area (km^2^)of the 70 Great Barrier Reef World Heritage Area bioregions, and the proportion (%) of each bioregion that was trawled each year between 2001 and 2009.
(DOCX)
