# Supplementary figures and images for: Interactions between a Trawl Fishery and Spatial Closures for Biodiversity Conservation in the Great Barrier Reef World Heritage Area, Australia
Source: PLoS One. 2011 Jun 13;6(6):e21094. doi: 10.1371/journal.pone.0021094 (PMC3113906; doi:10.1371/journal.pone.0021094)

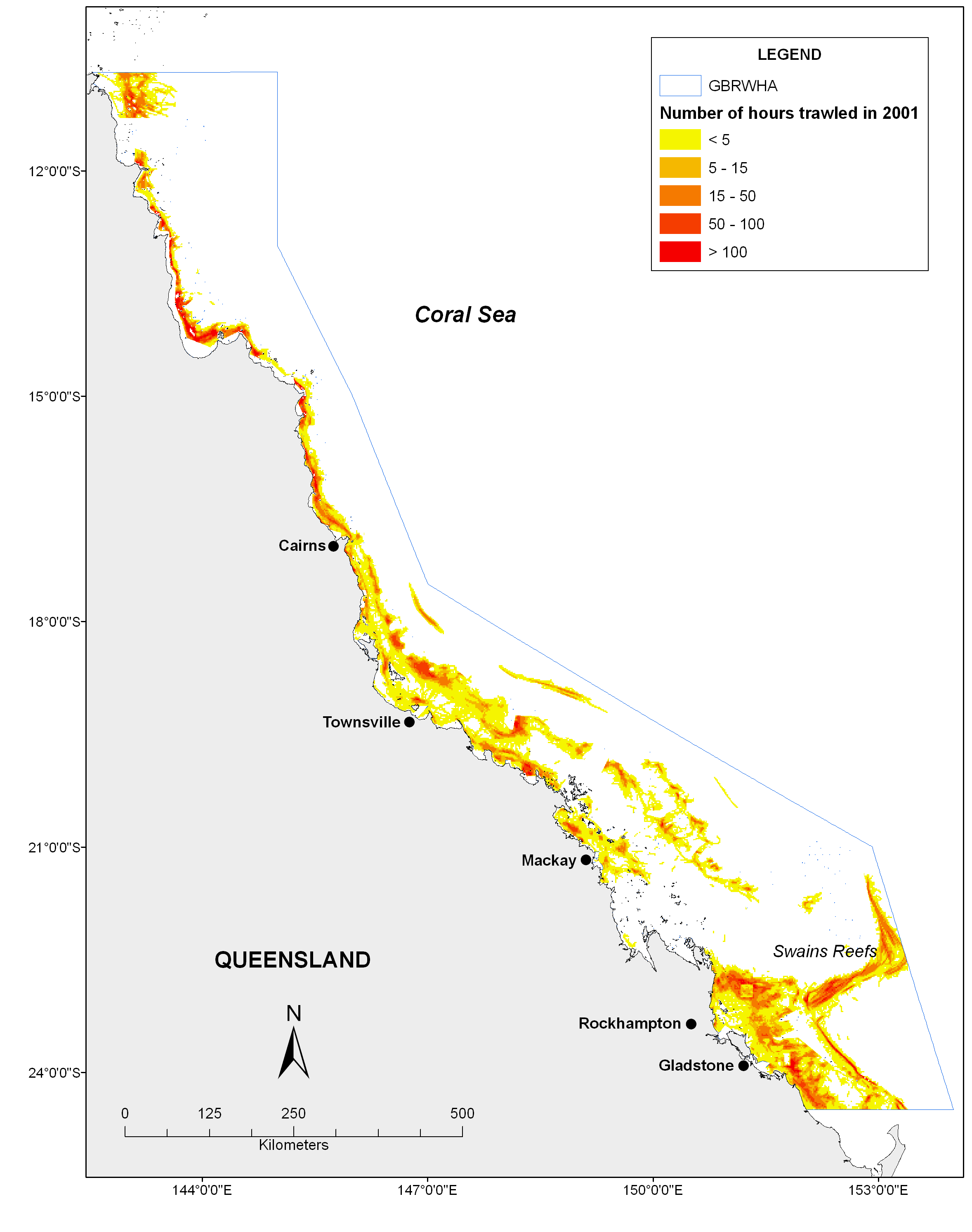

Supplement: Figure S1 — Trawl fishing effort (number of hours trawled per year) in 2001 [17] . (TIF) [file pone.0021094.s001.tif]

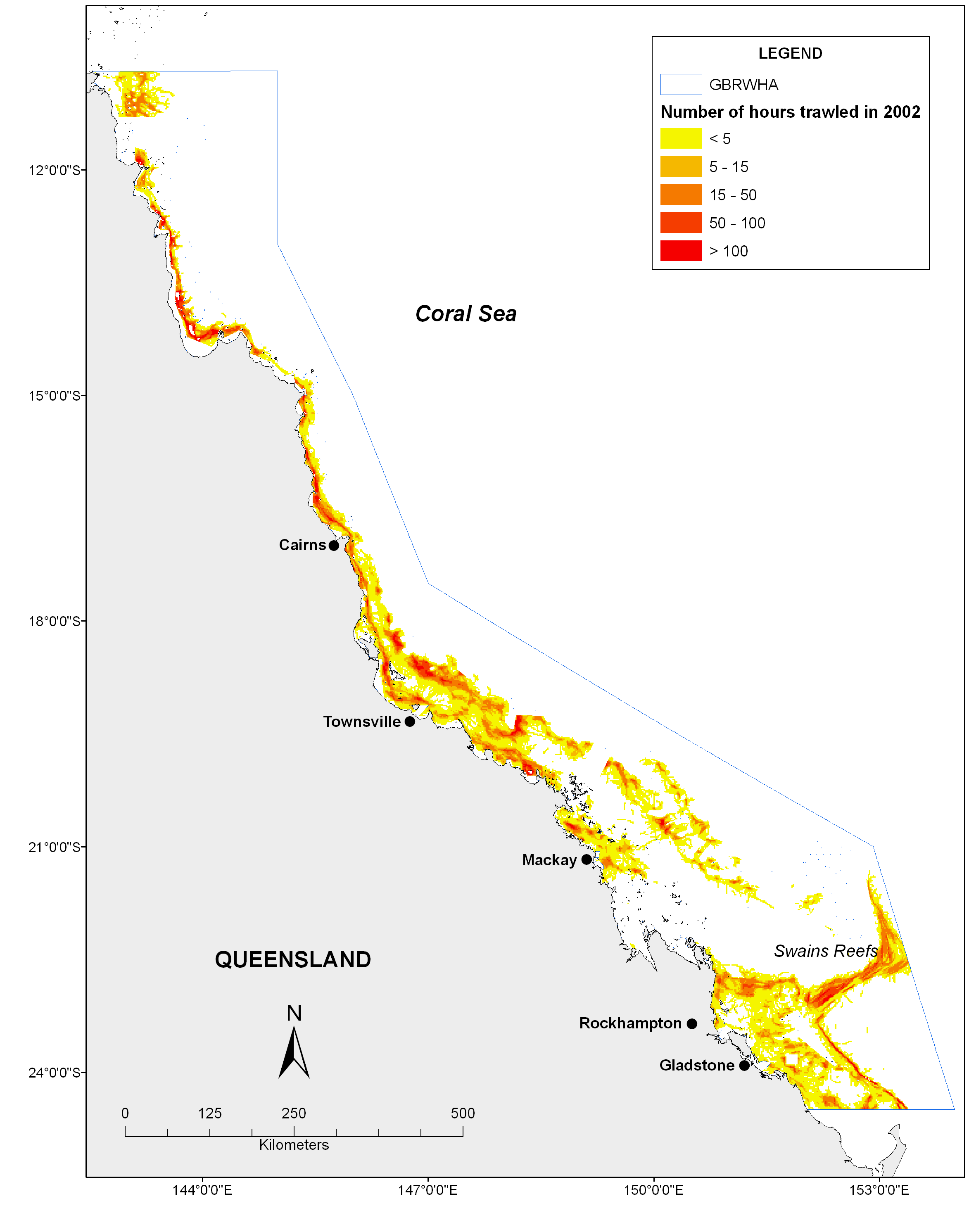

Supplement: Figure S2 — Trawl fishing effort (number of hours trawled per year) in 2002 [17] . (TIF) [file pone.0021094.s002.tif]

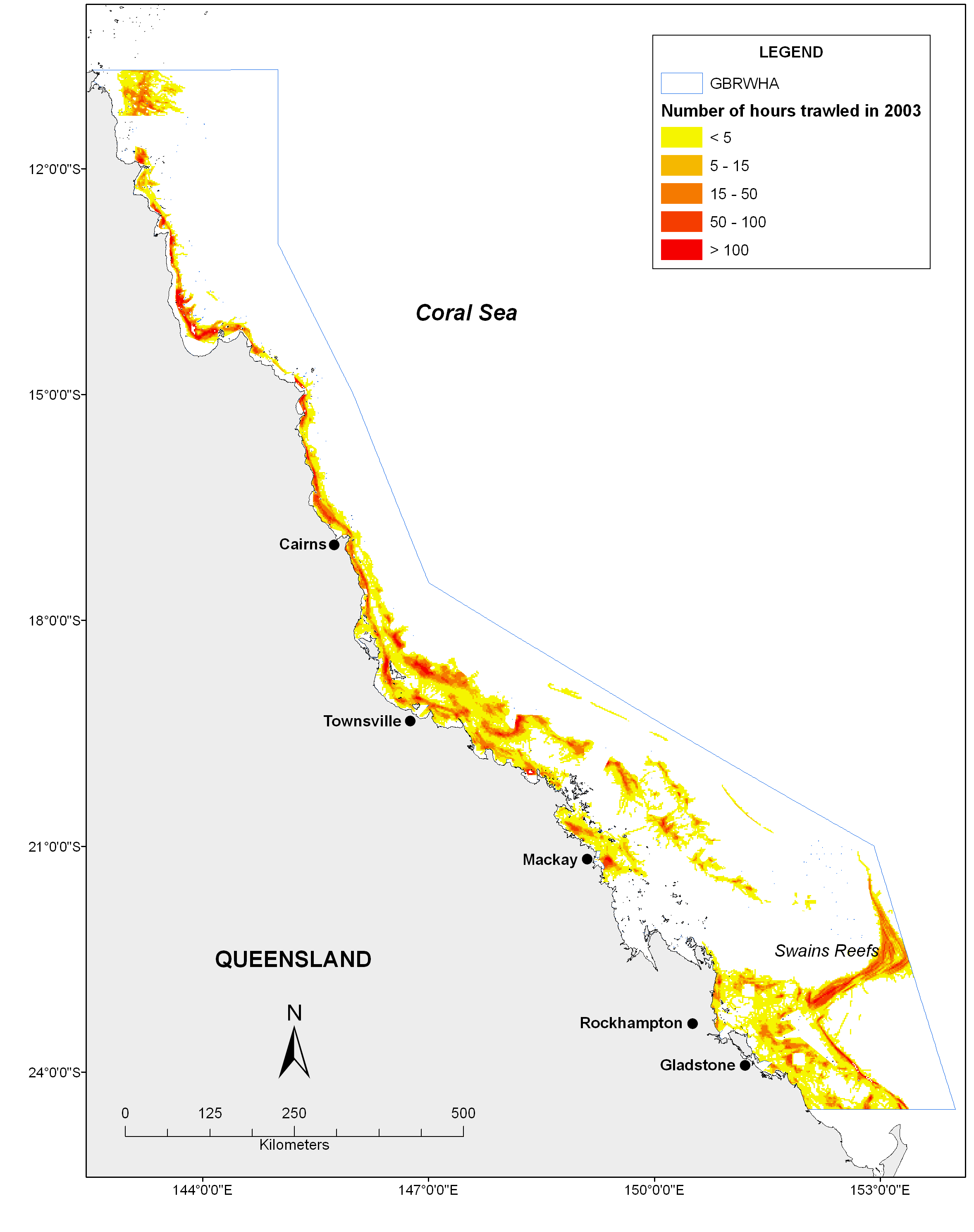

Supplement: Figure S3 — Trawl fishing effort (number of hours trawled per year) in 2003 [17] . (TIF) [file pone.0021094.s003.tif]

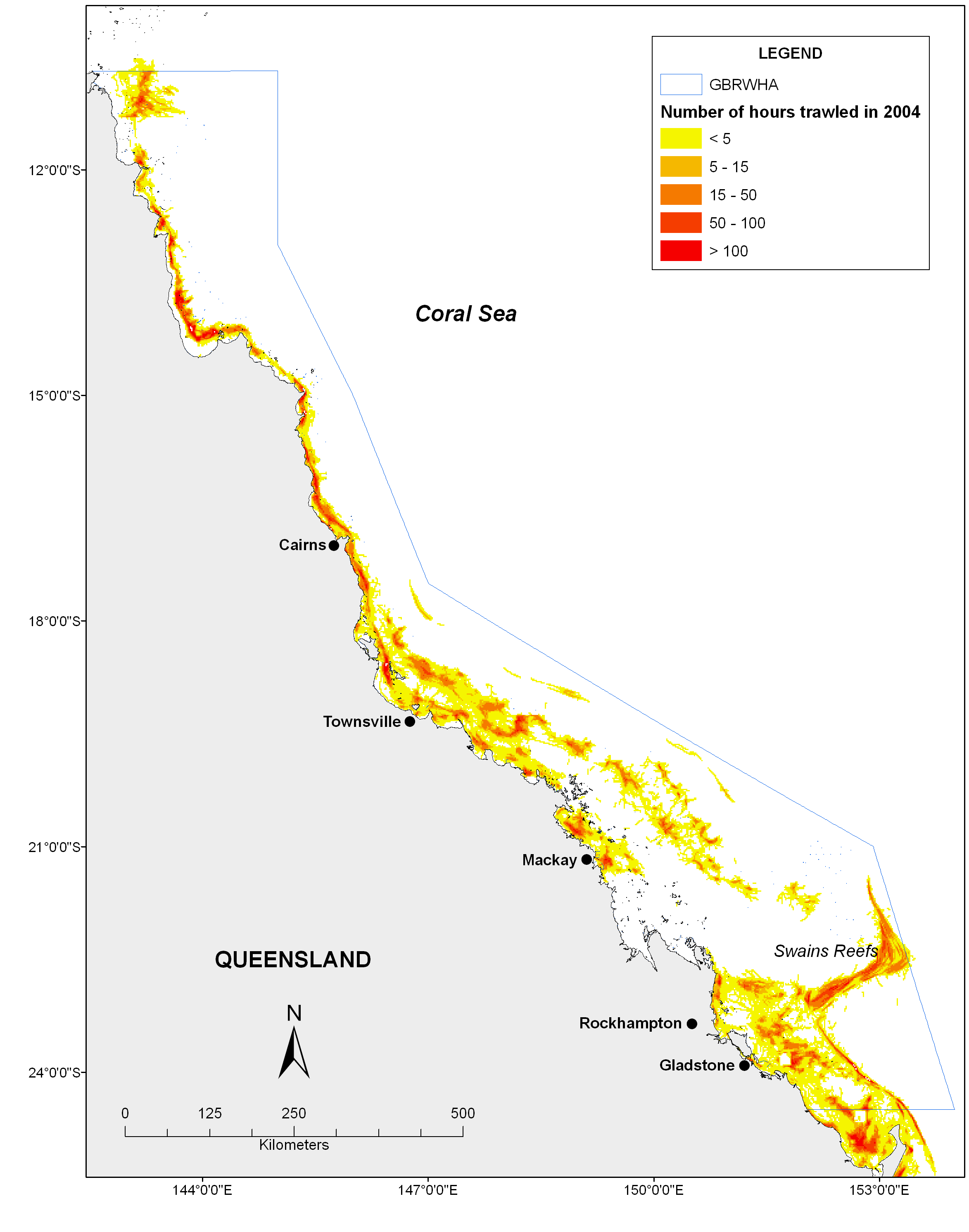

Supplement: Figure S4 — Trawl fishing effort (number of hours trawled per year) in 2004 [17] . (TIF) [file pone.0021094.s004.tif]

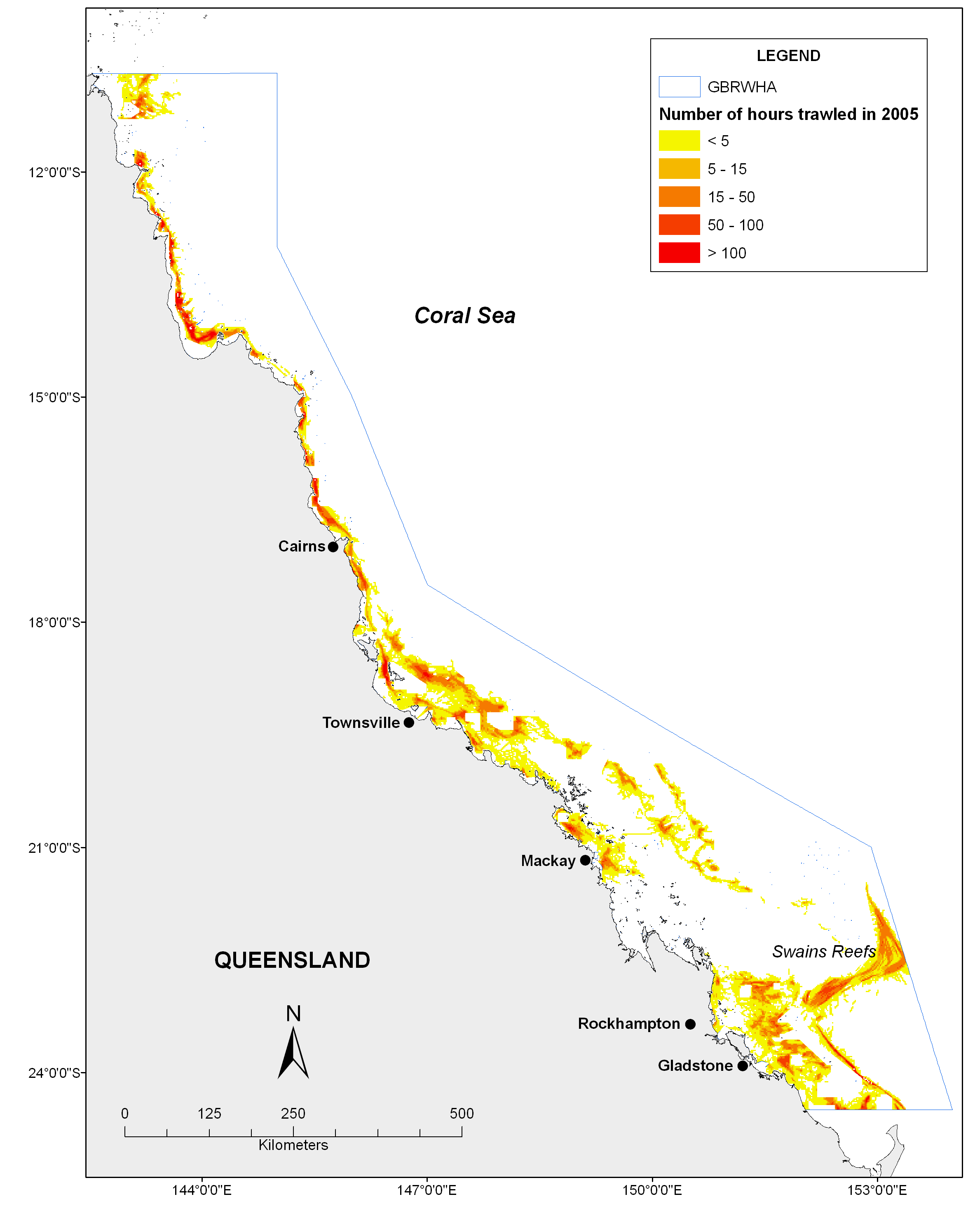

Supplement: Figure S5 — Trawl fishing effort (number of hours trawled per year) in 2005 [17] . (TIF) [file pone.0021094.s005.tif]

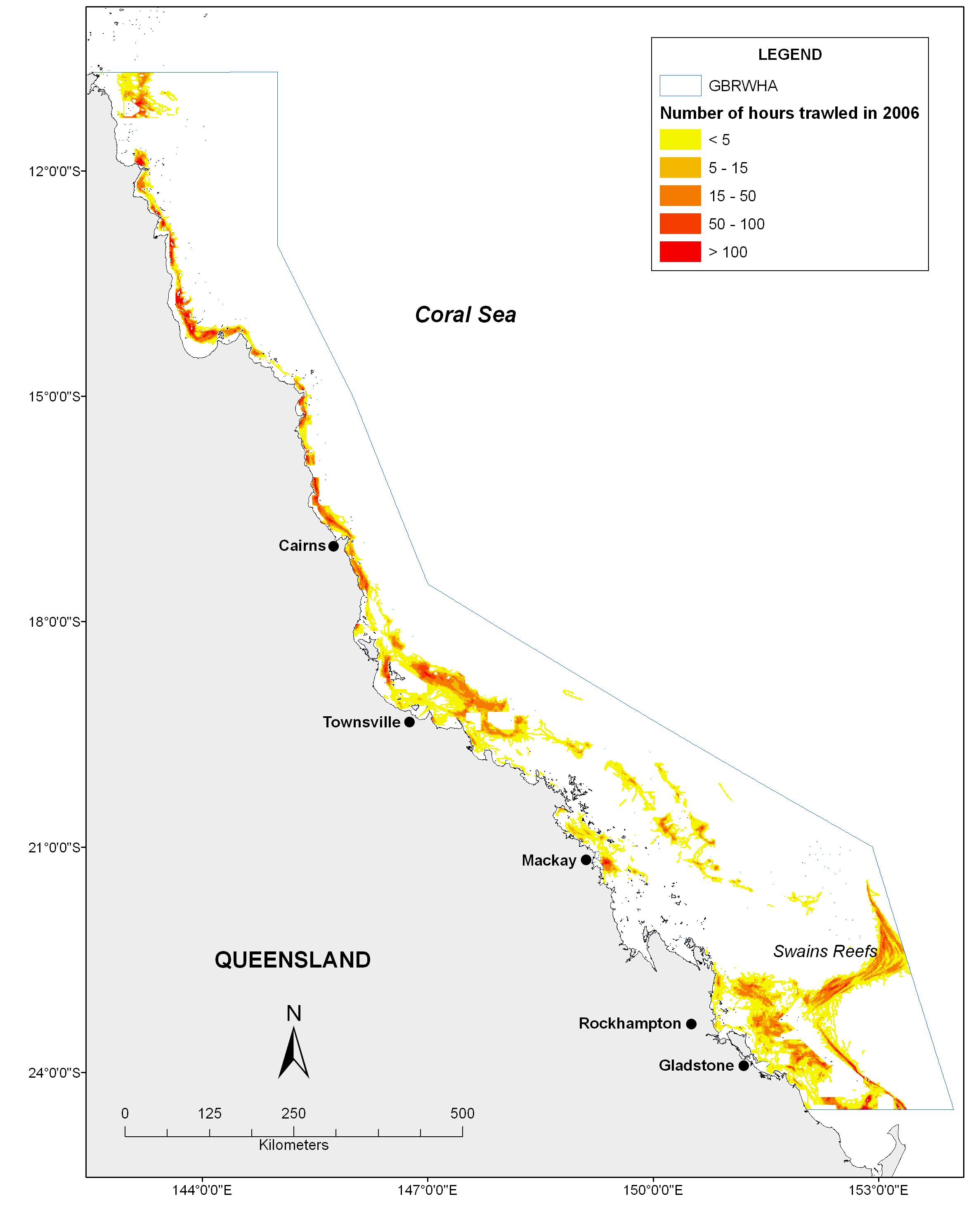

Supplement: Figure S6 — Trawl fishing effort (number of hours trawled per year) in 2006 [17] . (TIF) [file pone.0021094.s006.tif]

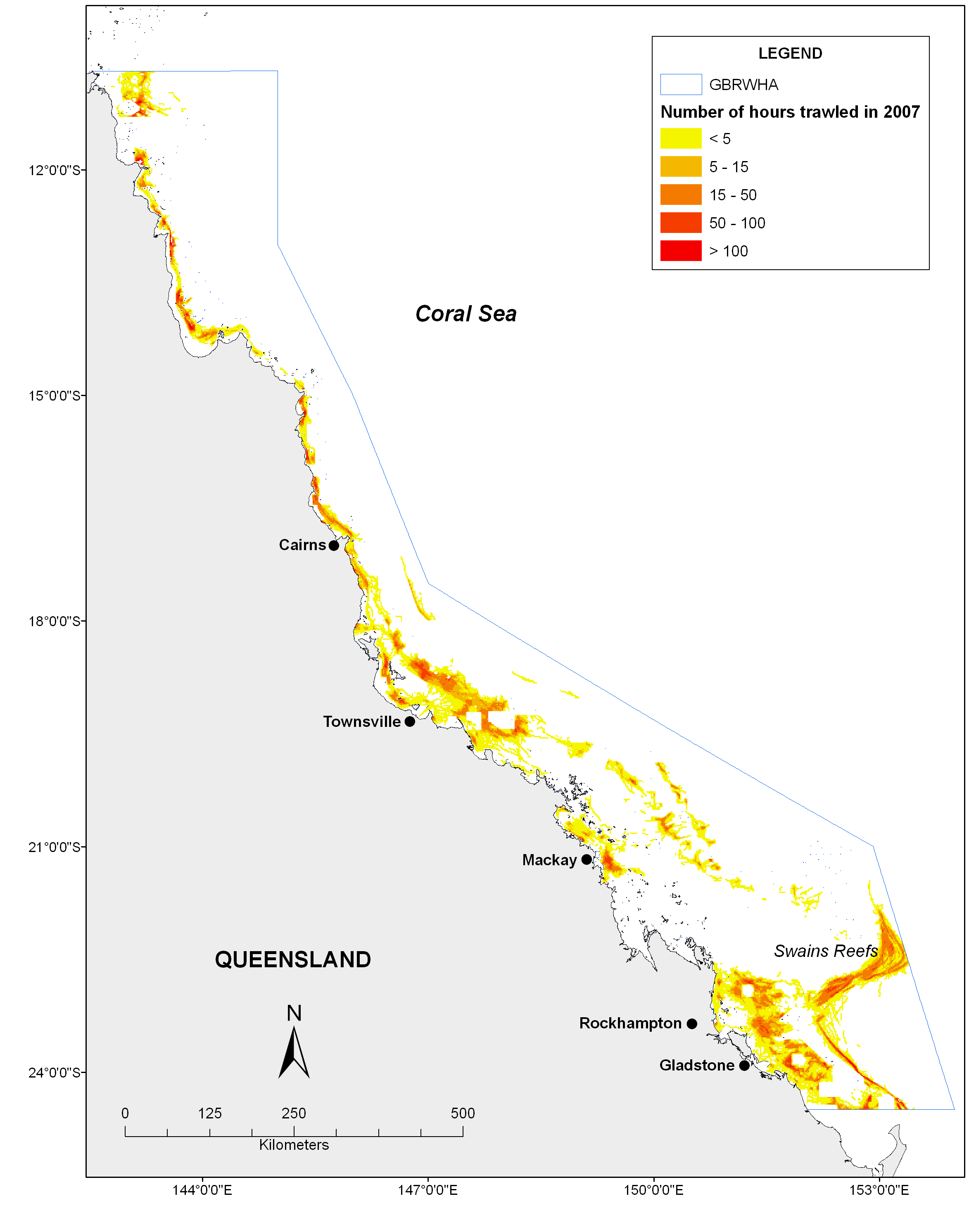

Supplement: Figure S7 — Trawl fishing effort (number of hours trawled per year) in 2007 [17] . (TIF) [file pone.0021094.s007.tif]

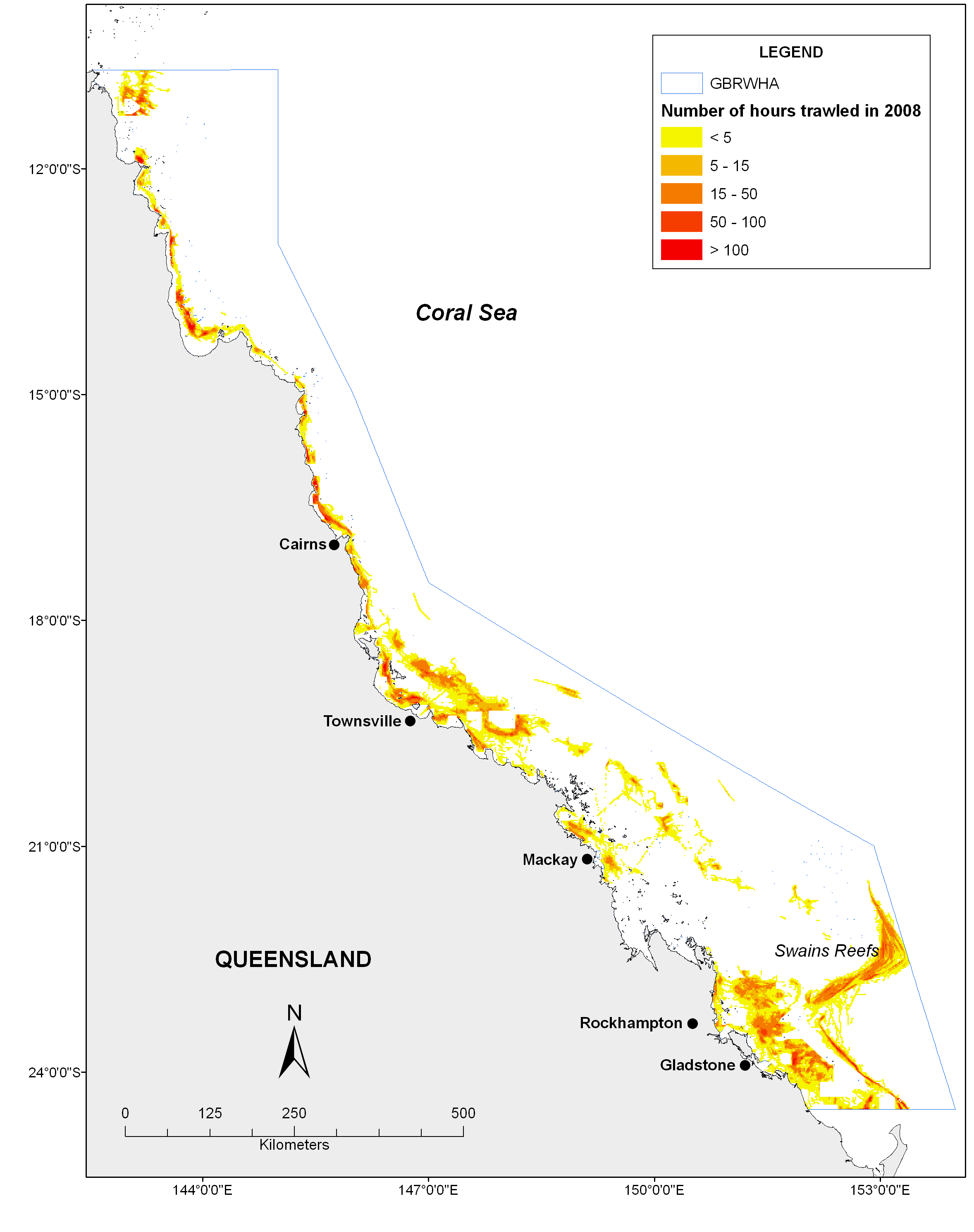

Supplement: Figure S8 — Trawl fishing effort (number of hours trawled per year) in 2008 [17] . (TIF) [file pone.0021094.s008.tif]

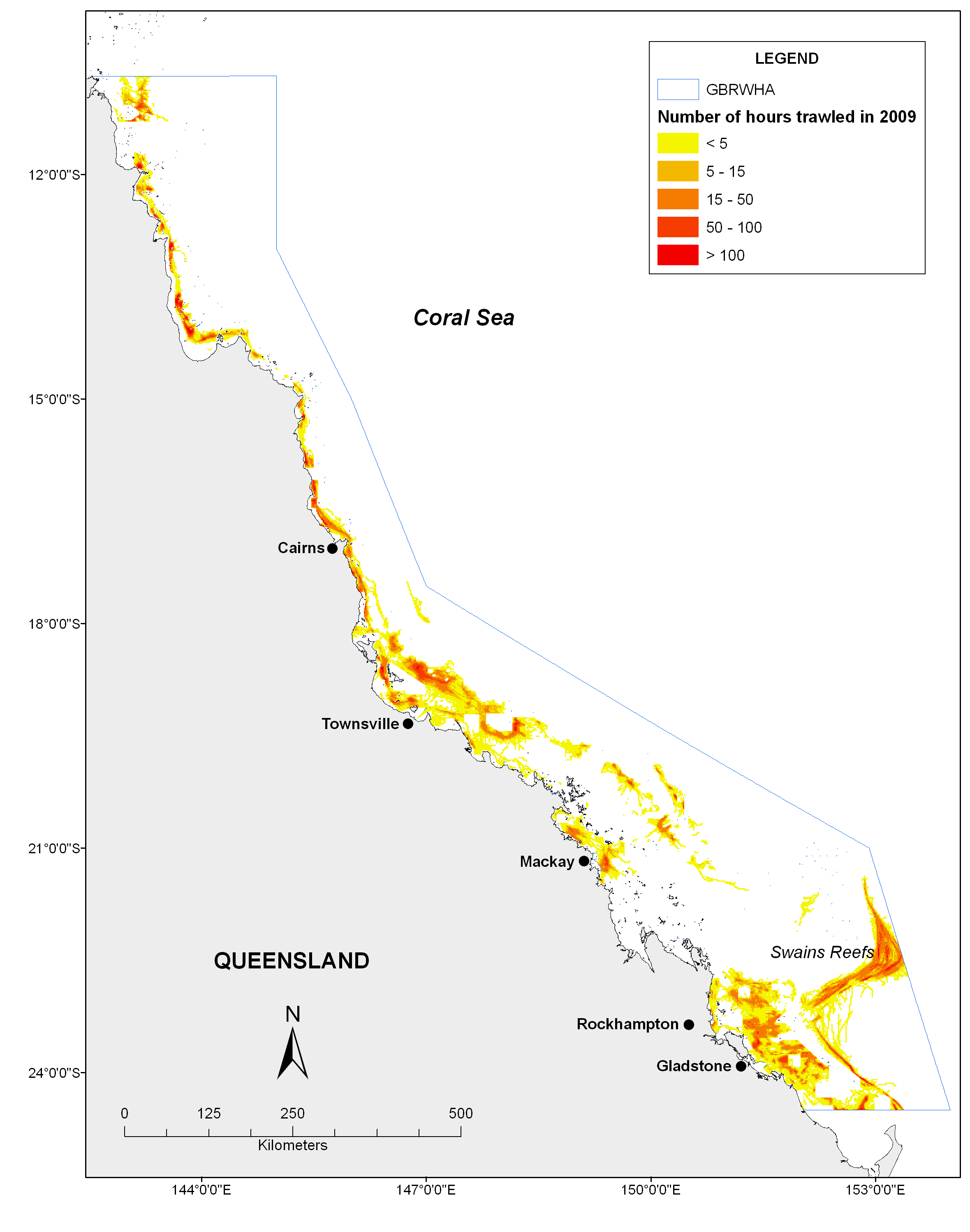

Supplement: Figure S9 — Trawl fishing effort (number of hours trawled per year) in 2009 [17] . (TIF) [file pone.0021094.s009.tif]
