# Supplementary material for: Interactions between a Trawl Fishery and Spatial Closures for Biodiversity Conservation in the Great Barrier Reef World Heritage Area, Australia
Source: PLoS One. 2011 Jun 13;6(6):e21094. doi: 10.1371/journal.pone.0021094 (PMC3113906; doi:10.1371/journal.pone.0021094)
Supplement: Table S1 — Area (km2)of the 70 Great Barrier Reef World Heritage Area bioregions, and the proportion (%) of each bioregion that was trawled each year between 2001 and 2009. (DOCX) [file pone.0021094.s010.docx]

| **Habitat** | **Bioregion** | **Area** | **2001** | **2002** | **2003** | **2004** | **2005** | **2006** | **2007** | **2008** | **2009** |
| --- | --- | --- | --- | --- | --- | --- | --- | --- | --- | --- | --- |
| Reef | Deltaic Reefs | 126 | 0.0 | 0.0 | 0.0 | 0.0 | 0.0 | 0.0 | 0.0 | 0.0 | 0.0 |
| Reef | Outer Barrier Reefs | 1,490 | 0.0 | 0.0 | 0.0 | 0.0 | 0.0 | 0.0 | 0.0 | 0.0 | 0.0 |
| Reef | Outer Shelf Reefs | 247 | 0.0 | 0.0 | 0.0 | 0.0 | 0.0 | 0.0 | 0.0 | 0.0 | 0.0 |
| Reef | Strong Tidal Outer Shelf Reefs | 214 | 0.0 | 0.0 | 0.0 | 0.0 | 0.0 | 0.0 | 0.0 | 0.0 | 0.0 |
| Reef | Far Northern Outer Mid Shelf Reefs | 1,443 | 0.2 | 1.4 | 1.9 | 0.1 | 0.7 | 0.7 | 0.0 | 0.0 | 0.0 |
| Reef | Torres Strait Influenced Mid Shelf Reefs | 204 | 3.5 | 16.3 | 7.8 | 9.0 | 1.2 | 1.2 | 4.1 | 1.6 | 2.0 |
| Reef | Far Northern Protected Mid Shelf Reefs and Shoals | 3,835 | 3.2 | 6.2 | 6.5 | 3.3 | 1.2 | 1.2 | 4.3 | 0.7 | 0.6 |
| Reef | Capricorn Bunker Outer Reefs | 389 | 10.9 | 11.4 | 8.0 | 16.6 | 1.3 | 1.3 | 1.4 | 1.2 | 0.8 |
| Reef | Capricorn Bunker Mid Shelf Reefs | 46 | 4.8 | 10.2 | 0.8 | 10.2 | 0.0 | 0.0 | 0.0 | 0.0 | 0.0 |
| Reef | Far Northern Open Lagoon Reefs | 1,081 | 0.7 | 0.7 | 0.7 | 0.8 | 0.9 | 0.9 | 1.0 | 0.9 | 0.9 |
| Reef | Coastal Far Northern Reefs | 278 | 0.4 | 0.7 | 0.7 | 0.7 | 0.0 | 0.0 | 0.1 | 0.0 | 0.0 |
| Reef | Coastal Northern Reefs | 89 | 7.3 | 7.5 | 6.2 | 6.6 | 0.2 | 0.2 | 0.2 | 0.2 | 0.2 |
| Reef | Coastal Central Reefs | 60 | 19.5 | 17.6 | 20.2 | 19.2 | 0.0 | 0.0 | 0.3 | 0.3 | 0.3 |
| Reef | Coastal Southern Reefs | 53 | 24.2 | 24.1 | 28.8 | 23.6 | 2.7 | 2.7 | 4.5 | 2.7 | 2.7 |
| Reef | High Tidal Fringing Reefs | 173 | 6.3 | 5.5 | 4.6 | 6.8 | 0.0 | 0.0 | 0.0 | 0.0 | 0.0 |
| Reef | Incipient Reefs | 143 | 0.0 | 0.0 | 0.0 | 0.0 | 0.0 | 0.0 | 0.0 | 0.0 | 0.0 |
| Reef | Tidal Mud Flat Reefs | 38 | 0.0 | 0.0 | 0.0 | 0.0 | 0.0 | 0.0 | 0.0 | 0.0 | 0.0 |
| Reef | Coastal Southern Fringing Reefs | 57 | 30.4 | 24.8 | 27.5 | 31.7 | 0.5 | 0.5 | 0.5 | 1.0 | 1.0 |
| Reef | Northern Open Lagoon Reefs | 131 | 1.0 | 1.1 | 0.9 | 1.6 | 0.0 | 0.0 | 0.0 | 0.0 | 0.0 |
| Reef | Central Open Lagoon Reefs | 2 | 59.8 | 59.8 | 59.8 | 55.2 | 0.0 | 0.0 | 0.0 | 0.0 | 0.0 |
| Reef | Sheltered Mid Shelf Reefs | 1,482 | 0.0 | 0.0 | 0.0 | 0.0 | 0.0 | 0.0 | 0.0 | 0.0 | 0.0 |
| Reef | Exposed Mid Shelf Reefs | 2,390 | 1.6 | 1.3 | 1.3 | 1.4 | 0.0 | 0.0 | 0.0 | 0.0 | 0.0 |
| Reef | High Continental Island Reefs | 102 | 1.0 | 1.0 | 1.0 | 1.0 | 0.0 | 0.0 | 0.0 | 0.0 | 0.0 |
| Reef | Strong Tidal Mid Shelf Reefs (East) | 260 | 0.0 | 0.0 | 0.0 | 0.0 | 0.0 | 0.0 | 0.0 | 0.0 | 0.0 |
| Reef | Hard Line Reefs | 1,571 | 0.0 | 0.0 | 0.0 | 0.0 | 0.0 | 0.0 | 0.0 | 0.0 | 0.0 |
| Reef | Strong Tidal Mid Shelf Reefs (West) | 2,104 | 0.3 | 0.1 | 0.2 | 0.2 | 0.0 | 0.0 | 0.0 | 0.0 | 0.0 |
| Reef | Strong Tidal Inner Mid Shelf Reefs | 923 | 0.0 | 0.1 | 0.0 | 0.1 | 0.0 | 0.0 | 0.1 | 0.1 | 0.0 |
| Reef | Swains Mid Reefs | 512 | 0.0 | 0.0 | 0.0 | 0.0 | 0.0 | 0.0 | 0.0 | 0.0 | 0.0 |
| Reef | Coral Sea Swains-Northern Reefs | 68 | 0.0 | 0.0 | 0.0 | 0.0 | 0.0 | 0.0 | 0.0 | 0.0 | 0.0 |
| Reef | Swains Outer Reefs | 141 | 0.0 | 0.0 | 0.0 | 0.0 | 0.0 | 0.0 | 0.0 | 0.0 | 0.0 |
| *Reef* | *Total* | *19,654* | *1.5* | *2.2* | *2.2* | *1.7* | *0.8* | *0.4* | *1.0* | *0.2* | *0.2* |
| Non-reef | Far Northern Coastal Strip | 2,771 | 9.1 | 9.1 | 8.9 | 9.6 | 7.1 | 7.1 | 7.9 | 7.1 | 7.0 |
| Non-reef | High Nutrients Coastal Strip | 17,166 | 47.2 | 47.5 | 46.7 | 48.4 | 31.0 | 31.0 | 35.6 | 32.1 | 35.1 |
| Non-reef | Inshore Terrigenous Sands | 6,318 | 11.8 | 11.7 | 9.7 | 10.3 | 10.3 | 10.3 | 11.3 | 8.4 | 8.8 |
| Non-reef | Inshore Muddy Lagoon | 8,915 | 54.5 | 51.5 | 49.6 | 57.1 | 41.5 | 41.5 | 44.5 | 42.7 | 41.7 |
| Non-reef | Inner Shelf Seagrass | 7,464 | 81.3 | 78.4 | 69.0 | 83.0 | 52.9 | 52.9 | 52.5 | 49.0 | 45.2 |
| Non-reef | Inner Mid Shelf Lagoon | 9,830 | 88.0 | 87.9 | 80.5 | 79.6 | 63.4 | 63.4 | 62.7 | 62.2 | 57.7 |
| Non-reef | Inner Shelf Lagoon Continental Islands | 14,644 | 25.2 | 22.2 | 20.4 | 24.4 | 12.7 | 12.7 | 16.6 | 12.5 | 9.5 |
| Non-reef | Mid Shelf Lagoon | 25,151 | 27.5 | 24.9 | 24.0 | 23.6 | 13.7 | 13.7 | 19.8 | 12.9 | 13.1 |
| Non-reef | Capricorn Bunker Lagoon | 16,718 | 71.0 | 68.1 | 70.1 | 82.6 | 63.8 | 63.8 | 59.3 | 63.9 | 63.3 |
| Non-reef | Mid Shelf Inter Reef - Seagrass | 5,522 | 41.2 | 46.4 | 42.3 | 42.8 | 29.4 | 29.4 | 35.6 | 25.5 | 26.0 |
| Non-reef | Mid Shelf Inter Reef | 2,838 | 6.0 | 27.6 | 18.5 | 12.0 | 3.6 | 3.6 | 10.2 | 0.8 | 2.1 |
| Non-reef | Outer Shelf Lagoon | 2,325 | 0.0 | 0.0 | 0.0 | 0.0 | 0.0 | 0.0 | 0.0 | 0.0 | 0.0 |
| Non-reef | Halimeda Banks - Some Coral | 740 | 0.0 | 0.0 | 0.0 | 0.0 | 0.0 | 0.0 | 0.0 | 0.0 | 0.0 |
| Non-reef | Mid Shelf Sandy Inter Reef | 1,339 | 26.8 | 29.7 | 27.6 | 28.3 | 17.9 | 17.9 | 25.0 | 17.8 | 20.2 |
| Non-reef | Halimeda Banks | 1,586 | 0.3 | 3.4 | 0.0 | 2.4 | 0.2 | 0.2 | 0.0 | 1.4 | 0.0 |
| Non-reef | Princess Charlotte Bay Outer Shelf | 686 | 26.4 | 27.6 | 16.8 | 27.9 | 2.5 | 2.5 | 11.8 | 5.7 | 4.3 |
| Non-reef | Princess Charlotte Bay | 1,443 | 39.3 | 37.0 | 35.6 | 39.6 | 34.1 | 34.1 | 38.8 | 26.1 | 30.2 |
| Non-reef | Outer Shelf Algae and Seagrass | 1,452 | 0.0 | 0.0 | 0.0 | 0.1 | 0.0 | 0.0 | 0.0 | 0.0 | 0.0 |
| Non-reef | Outer Shelf Seagrass | 5,016 | 6.6 | 5.8 | 3.3 | 8.3 | 0.6 | 0.6 | 1.2 | 1.2 | 0.5 |
| Non-reef | Outer Shelf Inter Reef - Central | 11,693 | 24.4 | 22.0 | 19.8 | 23.0 | 16.0 | 16.0 | 17.8 | 16.5 | 15.0 |
| Non-reef | Outer Shelf Inter Reef - Southern | 17,755 | 31.8 | 28.3 | 30.1 | 28.8 | 19.1 | 19.1 | 22.5 | 15.9 | 14.8 |
| Non-reef | Swains Inter Reef | 14,422 | 3.5 | 1.8 | 7.2 | 3.5 | 4.7 | 4.7 | 2.3 | 2.7 | 3.4 |
| Non-reef | Mid Shelf Seagrass | 2,833 | 20.7 | 19.3 | 18.1 | 22.6 | 8.2 | 8.2 | 15.3 | 8.9 | 12.8 |
| Non-reef | Capricorn Bunker Banks | 2,375 | 61.7 | 50.3 | 47.3 | 63.6 | 36.5 | 36.5 | 52.7 | 31.9 | 36.4 |
| Non-reef | Capricorn Trough | 5,821 | 9.4 | 6.9 | 7.5 | 8.5 | 4.2 | 4.2 | 6.7 | 3.0 | 4.2 |
| Non-reef | Eastern Plateau | 14,300 | 0.0 | 0.0 | 0.0 | 0.0 | 0.0 | 0.0 | 0.0 | 0.0 | 0.0 |
| Non-reef | Steep Slope | 8,790 | 0.0 | 0.1 | 0.0 | 0.1 | 0.0 | 0.0 | 0.0 | 0.0 | 0.0 |
| Non-reef | Queensland Trough | 5,285 | 0.0 | 0.0 | 0.0 | 0.0 | 0.0 | 0.0 | 0.0 | 0.0 | 0.0 |
| Non-reef | Intermediate Broad Slope | 2,745 | 0.0 | 0.0 | 0.0 | 0.0 | 1.0 | 1.0 | 0.0 | 0.0 | 0.0 |
| Non-reef | Eastern Pelagic Platform | 2,411 | 0.0 | 0.0 | 0.0 | 0.0 | 0.0 | 0.0 | 0.0 | 0.0 | 0.0 |
| Non-reef | Western Pelagic Platform | 17,699 | 0.0 | 3.9 | 7.3 | 10.1 | 3.8 | 3.8 | 0.0 | 2.1 | 3.5 |
| Non-reef | Terraces | 10,314 | 21.5 | 22.5 | 19.8 | 21.7 | 17.4 | 17.4 | 20.3 | 18.0 | 18.9 |
| *Non-reef* | *Total* | *248,365* | *27.7* | *27.0* | *26.3* | *28.6* | *24.0* | *19.5* | *21.0* | *18.6* | *18.5* |
| Deep | Far Northern Offshelf | 23,716 | 0.0 | 0.0 | 0.0 | 0.0 | 0.0 | 0.0 | 0.0 | 0.0 | 0.0 |
| Deep | Offshelf Queensland Trough | 5,476 | 0.0 | 0.0 | 0.0 | 0.0 | 0.0 | 0.0 | 0.0 | 0.0 | 0.0 |
| Deep | Outer Far Northern Inter Reef | 817 | 0.0 | 0.0 | 0.0 | 0.0 | 0.0 | 0.0 | 0.0 | 0.0 | 0.0 |
| Deep | Capricorn Bunker Inter Reef | 1,727 | 25.1 | 28.0 | 26.0 | 27.4 | 10.5 | 10.5 | 11.7 | 11.1 | 11.1 |
| Deep | Outer Central Inter Reef | 5,030 | 0.0 | 0.0 | 1.6 | 4.5 | 1.5 | 1.5 | 0.0 | 0.0 | 0.0 |
| Deep | Central Offshelf | 4,673 | 0.0 | 0.0 | 0.0 | 0.0 | 0.0 | 0.0 | 0.0 | 0.0 | 0.0 |
| Deep | Central Inter Reef | 9,613 | 1.4 | 1.2 | 1.0 | 1.0 | 0.6 | 0.6 | 0.9 | 0.5 | 0.3 |
| Deep | Southern Embayment | 29,486 | 23.9 | 23.2 | 22.1 | 23.7 | 23.5 | 23.5 | 23.0 | 25.9 | 23.5 |
| *Deep* | *Total* | *80,538* | *9.5* | *9.2* | *8.8* | *9.7* | *8.9* | *9.0* | *8.8* | *9.8* | *8.9* |
